# Creep Analysis of Bamboo Composite for Structural Applications

**DOI:** 10.3390/polym15030711

**Published:** 2023-01-31

**Authors:** Hayden Zanker, Ali Rajabipour, Dongsheng Huang, Milad Bazli, Siyuan Tang, Zhaoyan Cui, Jia Zhu, Joel Kennaway, Luis Herrera Diaz

**Affiliations:** 1 College of Engineering, IT & Environment, Charles Darwin University, Darwin 0801, Australia; 2 National Engineering Research Center of Biomaterials, Nanjing Forestry University, Nanjing 210037, China; 3 School of Mechanical and Mining Engineering, The University of Queensland, Brisbane 4000, Australia

**Keywords:** creep, fibre, bamboo composite

## Abstract

The present study investigates the phenomena of creep in a bamboo composite. The material was tested under tensile and compressive loading and simulated in finite element analysis software to estimate the creep coefficients. The presented findings have displayed the material’s propensity to fail at loads lower than the recorded ultimate strength, as early as 65% of this strength within 100 h, showing the importance of considering creep when designing structural components. Larger resistance to creep was observed under tensile stresses. Coefficients of the time-hardening creep model were estimated, which were found to be different under compression and tension. The findings provide insight into the reliable strength value of the Bamboo Composite. They could be also essential in estimating the long-term deflations in Bamboo Composite structures.

## 1. Introduction

The need for environmentally friendly engineered materials is increasing parallel to the environmental concerns and states’ legislation on sustainability [1,2]. With this need arrives the potential for greater utilisation of natural materials as alternatives to man-made solutions. An example of this, and the partial subject of this study, is natural fibre reinforced composites (NFRC) as alternatives to synthetic fibre reinforced composites (SFRC), such as the more commonly utilised glass or carbon fibre reinforced polymers. The use of natural composites is gaining more momentum in different industries while still structural applications of NFRC is under researched [3,4,5,6,7]. Despite this increase in use creep characteristics of NFRCs are note well studied yet [8,9,10]. Given the varied nature of strength characteristics in natural fibres and their composites, the importance of testing each unique composite profile before structural utilisation cannot be understated. In this study, an analysis of the creep characteristics of a parallel strand bamboo (PSB) composite will be investigated through experimentally creep testing and by utilising a simulation model in the finite element analysis (FEA) software, ANSYS Workbench.

It is known from a review of the literature that NFRC’s are especially susceptible to moisture ingress due to the hydrophilic nature of hemicellulose and that an increase in moisture content is likely to impact strength and modulus characteristics [4,11,12,13]. In addition to this, they also have a lower microbial and fire resistance, resulting in lower durability [10,14,15,16]. This susceptibility results in the material’s decreased strength and stiffness, likely making it more susceptible to the creep strain [16,17,18,19].

The PSB composite investigated herein contains longitudinally aligned fibres from the Phyllostachys species impregnated with a phenolic resin, resulting in an assumed transversely isotropic material with the greatest strength in tensile loading parallel to the fibre orientation [20]. The material thus far has been investigated under short-term loading and appears to present suitable characteristics for utilisation in structural applications. To better understand the potential weaknesses of the material, it is essential to investigate the effect of long-term loading and if this loading results in a significant reduction in the capacity of the material.

Literary investigations into the creep characteristics of some NFRC’s have shown that the strength capacities of this material decrease under loading much lower than the maximum capacity [21]. This decrease in strength is due to creep, the permanent deflection due to long-term sustained loading on a material [22,23]. Creep contains three regions: primary, secondary and tertiary. The present study will address primary and secondary creep, as tertiary creep typically corresponds with imminent failure [22]. Primary creep, unlike tertiary, refers to the region relatively soon after loading where elastic loads are redistributed and the strain rate decreases [22,24]. Secondary falls between the two and typically corresponds with a constant strain rate until tertiary creep commences. This process can be visualised in fibre reinforced composites through various micro and macroscopic phenomena. During secondary creep, polymer chains are provided energy by introducing a temperature or stress increase, with this energy allowing for barriers of motion to be overcome, enabling the chains to reach a new preferable energy state [25]. This process allows for the potential slippage of microfibrils and, given large enough loads, the permanent decrease in microfibrillar angle, resulting in increased stiffness after removal of loads [26,27]. Macroscopically, creep can also be witnessed through the cracking of the polymer matrix and a debonding between fibres and matrix [9]. Given that bonding between matrix and fibres is largely responsible for the strength capabilities of the composite, decreasing strength after long-term loading is unsurprising [4,28].

In bamboo composite, both fibres and the matrix could individually creep, but the composite creep is often assumed to be the same on the fibre and composite [22]. Studies show that the structural performance of the bamboo composite is largely influenced by moisture and creep [29]. This is due to the viscoelastic nature of composite, which has introduced many challenges to the structural design despite the advantages they offer [30]. While creep behaviour is a major challenge in using natural composites, the literature in this area is limited. One reason could be the long-term effect of creep over the structure’s service life of the structure, and thus, direct measurement of creep could take decades. In this regard, accelerated tests [30,31,32] have been proposed to predict the long-term creep behaviour of composites [33,34]. Further, mathematical models have been developed to predict long-term creep behaviour based on short-term test results [35,36].

Different creep models could be employed to model the long-term behaviour of bamboo composite when considered as a structural material. Creep models generally need a set of input parameters that could be estimated based on the experimental tests. These creep models could be used in simulation packages to estimate the creep behaviour of a structure made of PSB. In this study, such parameters are estimated according to the experimental results. While bamboo composite is deemed a promising sustainable alternative for some of the conventional construction materials, the creep parameters of this material have not been investigated to the best of the authors’ knowledge. These parameters are fundamental in the long-term design of structures and could be used in developing design standards and guidelines for the structural design of bamboo composite structures. Further, the results in here allow for complex simulations and to identify the potential for PSB’s use as a structural material in non-residential applications. 

## 2. Methodology

In solving the problem of investigating the creep characteristics of the PSB composite presented here, a thematic review of the available study proceeded with an investigation of experimental creep strain data sourced from laboratory testing. Material manufacturing and testing were completed at Nanjing Forestry University (NJFU). The testing methodology was generated through reference to the relevant ASTM testing guidelines [37,38]. 

ANSYS Workbench was utilised for simulation of the material, resulting in a need to determine the most suitable formula for mapping to the experimental material responses. The Norton-Bailey power law is a commonly applied model for determining primary and secondary creep. However, consultation of the available theories on the software presented an inability to use this common method since it does not allow for the mapping of creep within the primary response region and instead assumes a constant strain rate [39,40]. Therefore, the time hardening formulae were consulted as an alternative due to their ability to map the primary response of the material. ANSYS provides a variety of options, including Time Hardening (Primary), Generalised Time Hardening (Primary), Modified Time Hardening (Primary) & Time Hardening (Primary + Secondary). In this study, only the latter three were consulted due to the available testing data from experiments not aligning with the Time Hardening (Primary) formulation requirements. In each case, the equations contain a term allowing for dependency on temperature, referred to as the Arrhenius equation [41]. Due to the neglect of temperature effects in the testing of the material in the current study, the coefficient relating to this term was fixed at zero in each case. 

## 3. Material and Experimental Program and Results

PSB is a unidirectional fibre composite with fibres made from natural bamboo strands. The fibres are normally longer than 1500 mm. The matrix consists of an adhesive made from phenolic resin [42]. Fibres in PSB are extracted from 4–5-year-old Phyllostachys, a common bamboo species in South China. Bamboo culms are cut in strips of 2 m × 15 mm × 3 mm and dried at 80 °C prior to being flattened and impregnated with phenolic resin and finally pressed. The final product has an average density of 1100 kg/m^3^ [42].

**Figure 1:**
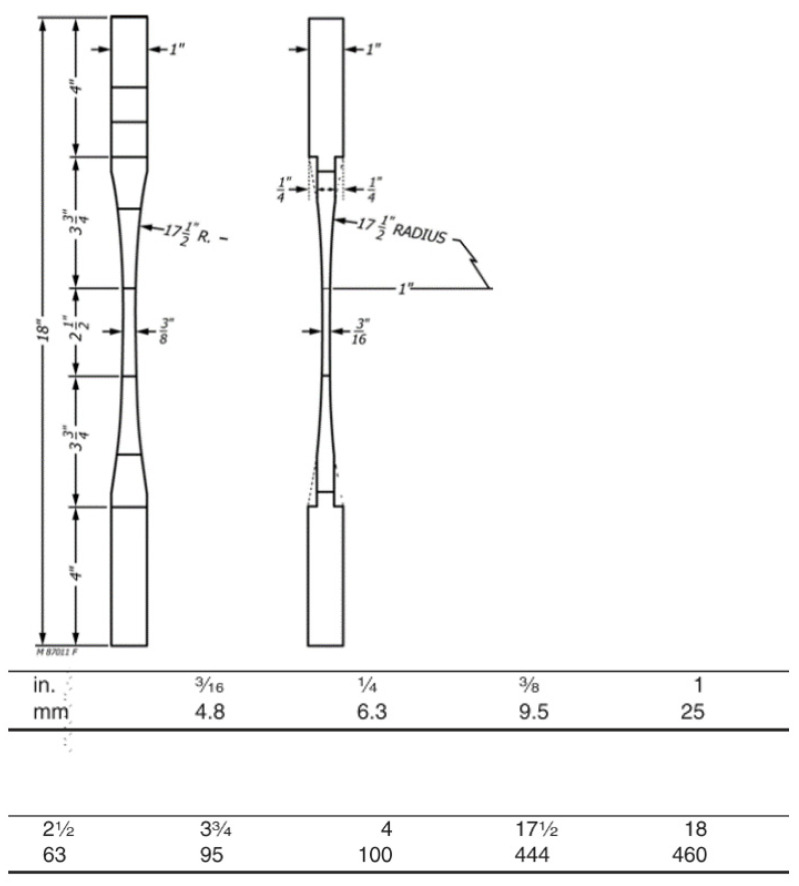
Tensile sample geometry as per ASTM D143 [38].

**Figure 2:**
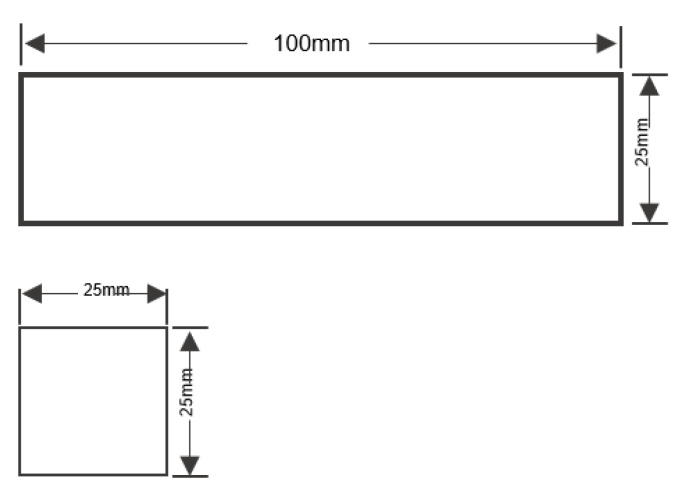
Compressive sample geometry as per ASTM D143 (mm).

In the selection of testing geometry, ASTM D143 and D695 were utilised for the tensile and compressive cases, respectively [37,38]. Figure 1 and Figure 2 display these geometries. In all cases, importance lies on the homogeneity of testing conditions and the assurance that all testing samples are conditioned at the testing conditions before loading, lest the samples be impacted by sorption hysteresis or a decrease in strength [13,14,43]. 

**Figure 3:**
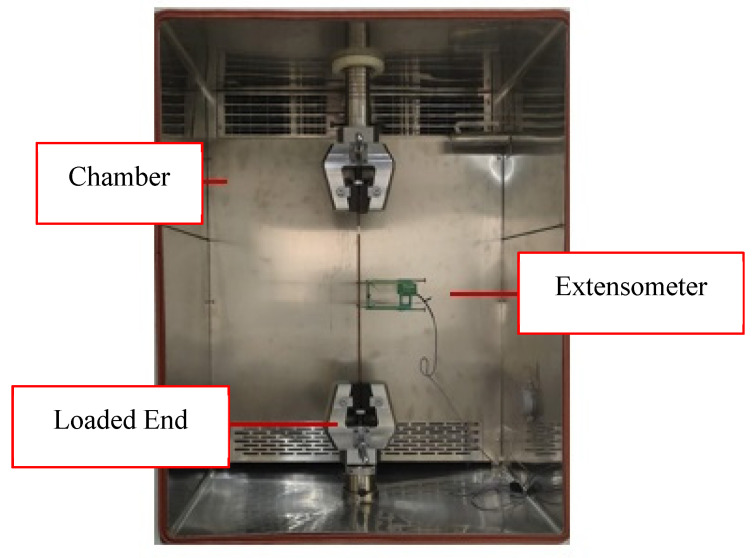
Tensile loading set-up.

**Figure 4:**
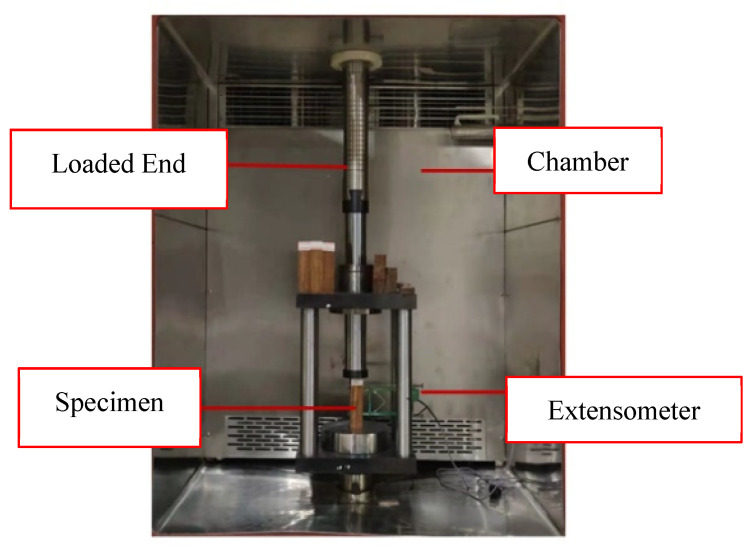
Compressive loading set-up.

Upon completion of conditioning, samples were placed within the testing environment with an attached mechanical extensometer allowing for automatic recording of the deformation under loading, as shown in Figure 3 and Figure 4. In both the tensile and compressive loading cases, the extensometer’s gauge length was 50 mm, allowing for conversion to directional strain as necessary. In total, 16 tensile and 13 compressive samples were investigated with distribution among force ratios displayed in Table 1. This testing was completed in ambient laboratory conditions.

**Figure 5:**
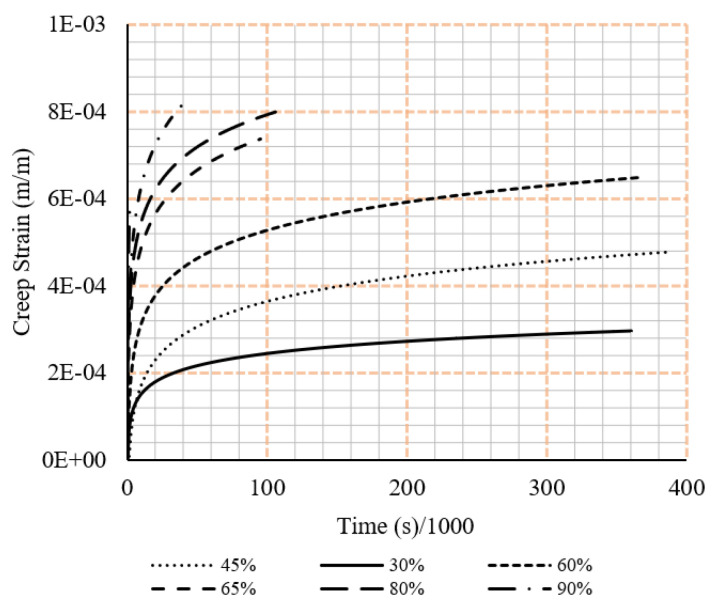
Tensile creep data from the experimental testing under different loading rations (ratio of sustained load to the ultimate load).

**Figure 6:**
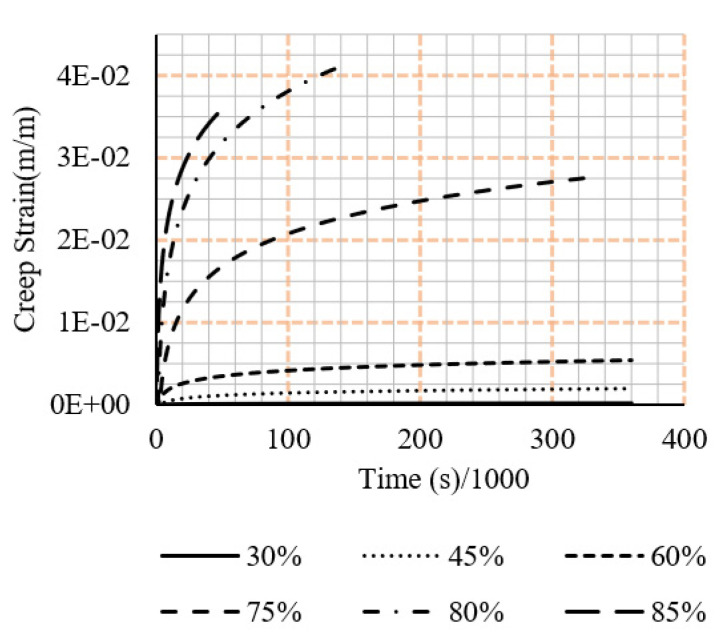
Compressive creep data from the experimental testing under different loading rations (ratio of sustained load to the ultimate load).

Along with this are the respective strain vs time graphs. In all cases, the displayed results exclude the unloading of the sample as the current study excludes a discussion on the relaxation effects of the samples. Creep tests results are shown in Figure 5 and Figure 6.

## 4. Simulation Results

An ANSYS numerical simulation model was built to re-produce the unconfined PSB connection experimental results and clarify experimental observations using finite element analysis software. The 2019R2 version of ANSYS was used.

ANSYS-R19 was used to find the set of input parameters that best fit the simulations and experimental results. Two creep models were considered for developing and calibrating the simulation models, which are shown in Equation (1)

Generalised Time Hardening:

(1) $$\dot{\varepsilon }=ft^{r}e^{\frac{-C_{6}}{T}}$$

(2) $$f=C_{1}\sigma +C_{2}\sigma^{2}+C_{3}\sigma^{3}$$

(3) $$r=C_{4}+C_{5}\sigma$$

In which:

$\dot{\varepsilon }:$ Rate of stain change by time.
t:  Time
T: Temperature

The simulation utilised SOLID186 finite elements with mesh conditions defined by the system. Time increment boundaries were allocated but were suitably spaced to allow for the system to select appropriate increments, ensuring the simulation did not exceed the creep limit ratio. As the calculation has time dependency, the selection of time increment is essential to ensure the convergence of simulations. Initially, the elastic simulation of the compressive specimen was completed through the utilisation of a complete model. This was then replaced with a quarter model utilising zeroed deformation at the internal faces of the material to decrease the computational burden of the simulation. Doing so resulted in an accurate stress distribution compared to the expected stresses and is validated under symmetric geometry and loading assumptions. Upon validation of the model elastically, generation of creep coefficients was necessary for application within the ANSYS Mechanical dashboard. Recommended methods for this process are available in the pre-existing literature, however the simplest and most efficient method determined was the use of the Mechanical APDL curve fitting procedure within the ANSYS software [39]. with the decreasing strain rate evident from the experimental data, the time hardening formulae were consulted for the curve mapping process.

PSB mechanical properties are defined through the previous study [20]. These properties are listed in Table 2 along with any further relevant defined parameters for each model. The material is reported to be transversely isotropic [42]. The Y axis, parallel to the fibre direction, displays larger strength and shear resistance and is the principal axis of loading in both the experiments and simulations. In all simulation cases, the temperature was left at ambient conditions. 

**Figure 7:**
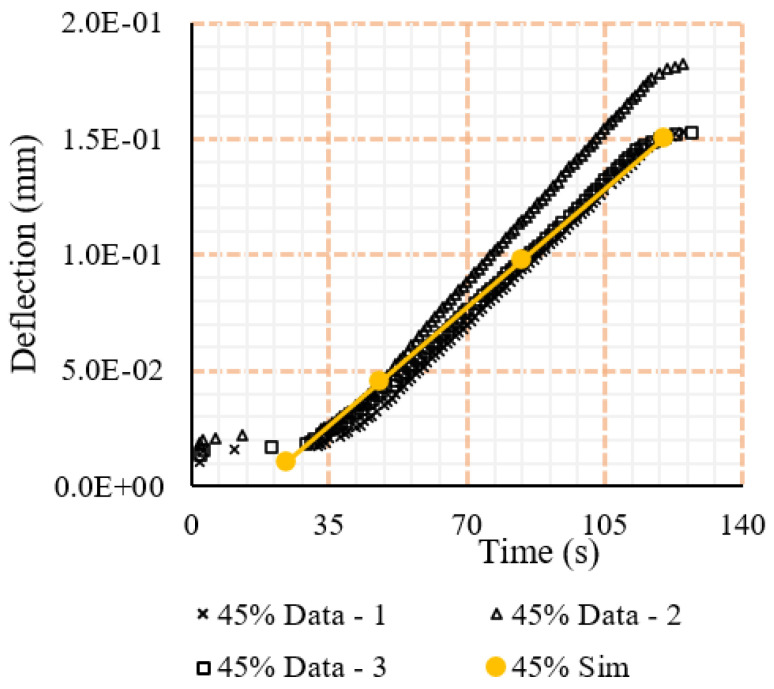
Elastic response comparison under 45% tensile loading (ratio of sustained load to the ultimate load) for trials and simulation.

To ensure calibration of the simulation models, the elastic deformation witnessed from the simulations was compared to that of the experimental equivalent to ensure the stress applied and response from the sample were similar. These comparisons showed that for the large majority of testing intervals, the response was largely the same (see Figure 7), however, for the larger force ratios, differences were seen between the two. This could be associated with the imperfection in the material such as fibre misalignment, which has been shown in the literature to have an effect on the recorded material properties [7,15,44]. 

**Figure 8:**
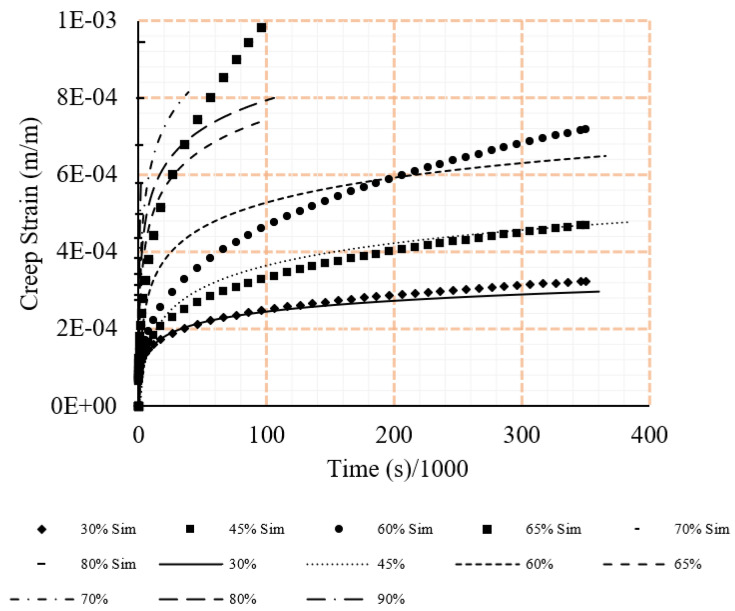
Comparison between experiment and simulation tensile strains under different loading rations (ratio of sustained load to the ultimate load).

All data from the tensile loading case inserted into the Mechanical APDL dashboard in a singular case. Doing so, resulted in a singular set of coefficients which, when utilised in simulations by inserting into the Engineering Data tab of the PSB, achieved accurate results. The coefficients and results from the simulations can be seen in Table 3 and Figure 8.

**Figure 9:**
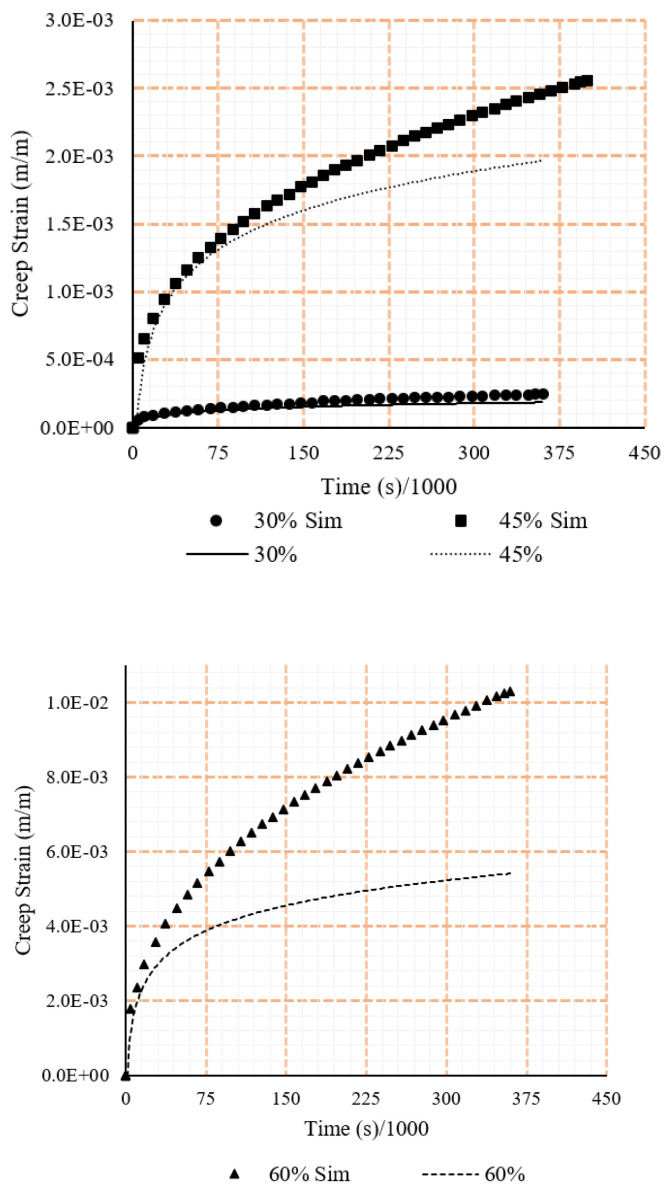
Comparison between experiment and simulation compressive strains under different loading rations (ratio of sustained load to the ultimate load).

A similar process was prescribed for the compressive loading scenario. However, it was witnessed from the tests that the difference in strain between samples in the experimental testing was much larger, including an approximate 33% difference for the 45% compressive iteration. To address the differences, a second iteration utilising data of higher strains at each force ratio was completed in an effort to more closely map the larger responses, allowing for a greater impression of maximum strains applicable at each force. This second iteration successfully displayed the upper bound of strains in each case while still largely remaining within the experiment’s witnessed strain regions. The coefficients and simulation results can be seen in Table 4 and Figure 9. As Figure 9 suggests, when stress was more than 45% of the ultimate stress, simulation results did not match the experimental results. 

## 5. Discussion 

It is important to note that although a variety of formulae are available for use, and many can appear valid, the software contains an assumption of constant strain rate (and, by default linearity) in the secondary creep choices. This means that in cases where the experiments may appear to be sufficiently long in timespan, a non-linear data curve will not be as closely represented by the non-linear options. This was experienced in the present study that although the data could be curve fitted to the Norton-Bailey Power Law outside of ANSYS Workbench, the returned creep coefficients resulted in a non-running simulation within the software.

The testing revealed the propensity of the material to fail at loads significantly below the recorded maximum strengths. Although strength characteristics of the material parallel to the alignment of fibres display significant strength under simple loading, a large portion of the recorded strength cannot be recruited in real world scenarios without resulting in failure over time. This presents whether strength comparisons between the composite and structural materials adequately compare the differences in the strength that can actually be recruited. A designer attempting to utilise calculated strains may include the use of a safety factor. However, one cannot be presented here without further testing on the environmental effects. Alternatively, the simulation can be utilised merely as a guide to the expected range of strains and for identifying regions of material likely to increase the chance of failure due to stress concentrations.

**Figure 10:**
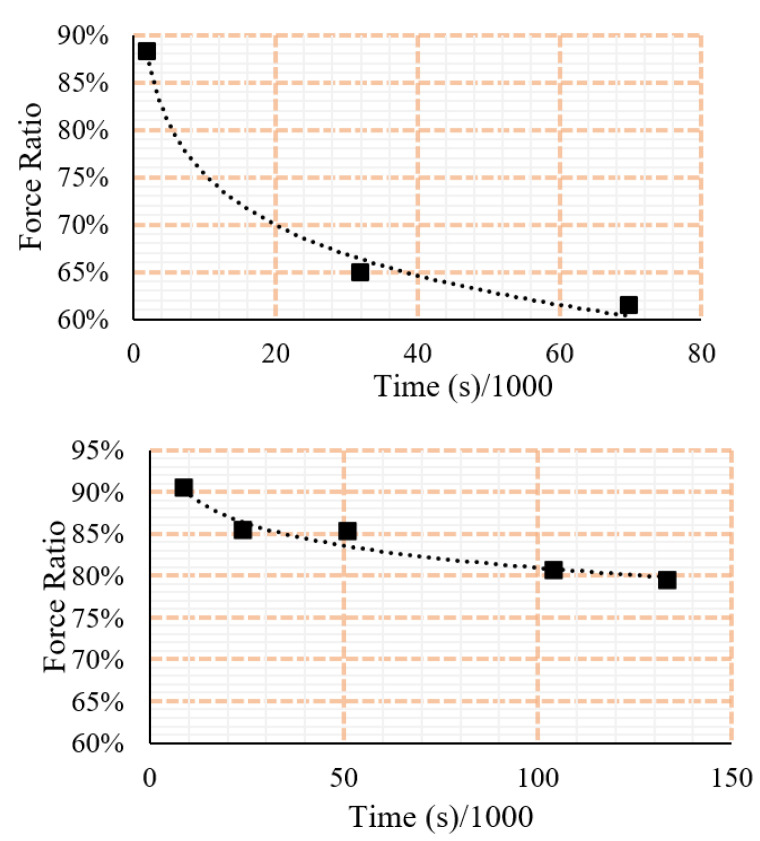
Changes of strength by time under different loading rations (ratio of sustained load to the ultimate load). (**Up**) failure curve under tensile loading and (**Down**) failure curve under compressive loading Please note that the scale is different in the two diagrams.

If PSB is expected to withstand long-term loading, a significate proportion of the available strength cannot be utilised. Figure 10 shows the decline of strength in PSB by time under tensile and compressive stresses. Compressive, in comparison to tensile loading, appears to have a larger region for satisfactory performance without failure occurring. This is inhibited, however, as the tensile capacity of the material is significantly larger than compressive, meaning the tensile capacity still appears larger. It is hypothesised that, due to the large comparative decrease in the tensile loading scenario, the difference is related to the interaction between fibre reinforcement and matrix. Specific mechanisms causing this may relate to the structure of the microfibrils in comparison to the polymer matrix and are outside the scope of the present study. 

Manufacture of the material may find benefit in future by utilising a multi-directional fibre alignment to allow for greater strength in the secondary and tertiary axes. However, this would come at the sacrifice of the strength of the primary axis. Doing this would also homogenise the response to long-term loading and would likely allow for the generation of a singular response curve for long-term loading. It may also be found from an investigation of such an alignment that the multi-directional fibre alignment mitigates failure modes such as cleavage. If completing this, the manufacturer should take note of the minimum recommended fibre sizes presented in the literature, with bast fibres typically in the 0.2–3 mm range and bamboo at either 4mm or a length to diameter ratio of 100 [7,45,46].

The material’s resistance to creep deformation is larger in the tensile loading cases when compared to the compressive iterations. This increased stiffness could result from the reinforcing fibres, with the lignin-carbohydrate matrix comprising the bamboo fibres having greater stiffness than the polymer matrix [47,48]. In compression, fibres are not fully recruited, and the stiffness is largely due to the surrounding polymer matrix [20,22,42]. This difference results in an inability to provide a singular estimation of creep strain in complex geometries in instances of both compressive and tensile stresses. Instead, only a region can be provided with this likely to encompass the true strains expected of the material. Although this is a clear limitation of the simulation process, there is still usefulness found in the simulations through the ability to identify areas of concern. This can include areas of stress concentration, such as around bolt holes or areas that appear to fail prematurely.

Future investigation may be completed on the development of a ‘Master Curve’. This has been presented in depth by Guedes [49] and allows for the superposition of creep data for varied loading and temperature cases under the assumption that in all cases the variables would not otherwise cause damage to the material. Investigating this would mitigate the need for further testing if it was found that the impact of temperature was consistent, however, the curve would be unable to superimpose reactions at larger stress levels as it has been shown in the present study that the reaction at these forces differs to the lower iterations.

## 6. Conclusions

Findings important to the analysis of the PSB composite as a structural material includes:

- The material fails at loads significantly below its recorded maximum strength due to creep, including at the 65% force ratio for tensile and at the 75% force ratio for compressive.
- Simulation accuracy decreases at higher force ratios possibly due to failure of the material and tertiary creep.
- The material has two distinct set of creep coefficients for tensile and compressive loading.
- A significant proportion (approx. 50%) of the maximum strength cannot be utilised in long-term loading due to the risk of failure.
- The results obtained here provide the safe load/capacity ratio in structural applications of BC. In this regard, the stress in BC is recommended to be kept under 50% of the ultimate strength for long-term application.
- The finding could also be used in numerical simulations to estimate long-term deflection in BC structures.
- Findings could be used in developing guidelines for the structural design of bamboo structures.
- Future investigations should quantify the impact of temperature and humidity increases on the long-term strength of the material, although it is known that this effect will be negative.

## Figures and Tables

**Table 1:** Number of tensile samples tested at each force ratio.

Force Ratio	Tensile	Compressive
30%	1	2
45%	3	2
60%	3	2
65%	2	-
70%	3	-
75%	-	1
80%	2	2
85%	-	2
90%	2	2

**Table 2:** Material data input into ANSYS corresponding to the geometry axis.

Property	X	Y	Z
Density	7850 kg/m^3^		
Modulus	4.35 GPa	16.3 GPa	4.35 GPa
Poisson Ratio	0.28		
Shear Modulus	3.8 GPa (XY)	4.5 GPa (YZ)	3.8 GPa (XZ)
Tensile Yield	5 MPa	128 MPa	5 MPa
Compressive Yield	45 MPa	84 MPa	45 MPa
Shear Yield	15 MPa (XY)	31 MPa (YZ)	15 MPa (XZ)

**Table 3:** Tensile Creep Coefficients from the Group Generalised Time Hardening simulations.

Coefficient	Value
$C_{1}$	$2.0997\times 10^{-12}$
$C_{2}$	$-5.428\times 10^{-20}$
$C_{3}$	$3.723\times 10^{-28}$
$C_{4}$	$7.208\times 10^{-2}$
$C_{5}$	$3.786\times 10^{-9}$
$C_{6}$ (Arrhenius Term)	0

**Table 4:** Compressive Creep Coefficients from the Group Generalised Time Hardening simulations.

Coefficient	Value
$C_{1}$	$7.685\times 10^{-13}$
$C_{2}$	$3.637\times 10^{-20}$
$C_{3}$	$-8.179\times 10^{-29}$
$C_{4}$	$2.502\times 10^{-1}$
$C_{5}$	$2.995\times 10^{-9}$
$C_{6}$ (Arrhenius Term)	0

## Data Availability

Upon request.
